# Predicting the impact of border control on malaria transmission: a simulated focal screen and treat campaign

**DOI:** 10.1186/s12936-015-0776-2

**Published:** 2015-07-12

**Authors:** Sheetal P Silal, Francesca Little, Karen I Barnes, Lisa J White

**Affiliations:** Department of Statistical Sciences, University of Cape Town, Rondebosch, Cape Town, 7700 South Africa; Division of Clinical Pharmacology, Department of Medicine, University of Cape Town, Observatory, Cape Town, 7925 South Africa; Mahidol-Oxford Tropical Medicine Research Unit, Mahidol University, Bangkok, Thailand; Nuffield Department of Clinical Medicine, Centre for Tropical Medicine, Churchill Hospital, University of Oxford, Oxford, UK

**Keywords:** Imported infections, Malaria, Elimination, Focal screen and treat

## Abstract

**Background:**

South Africa is one of many countries committed to malaria elimination with a target of 2018 and all malaria-endemic provinces, including Mpumalanga, are increasing efforts towards this ambitious goal. The reduction of imported infections is a vital element of an elimination strategy, particularly if a country is already experiencing high levels of imported infections. Border control of malaria is one tool that may be considered.

**Methods:**

A metapopulation, non-linear stochastic ordinary differential equation model is used to simulate malaria transmission in Mpumalanga and Maputo province, Mozambique (the source of the majority of imported infections) to predict the impact of a focal screen and treat campaign at the Mpumalanga–Maputo border. This campaign is simulated by nesting an individual-based model for the focal screen and treat campaign within the metapopulation transmission model.

**Results:**

The model predicts that such a campaign, simulated for different levels of resources, coverage and take-up rates with a variety of screening tools, will not eliminate malaria on its own, but will reduce transmission substantially. Making the campaign mandatory decreases transmission further though sub-patent infections are likely to remain undetected if the diagnostic tool is not adequately sensitive. Replacing screening and treating with mass drug administration results in substantially larger decreases as all (including sub-patent) infections are treated before movement into Mpumalanga.

**Conclusions:**

The reduction of imported cases will be vital to any future malaria control or elimination strategy. This simulation predicts that FSAT at the Mpumalanga–Maputo border will be unable to eliminate local malaria on its own, but may still play a key role in detecting and treating imported infections before they enter the country. Thus FSAT may form part of an integrated elimination strategy where a variety of interventions are employed together to achieve malaria elimination.

**Electronic supplementary material:**

The online version of this article (doi:10.1186/s12936-015-0776-2) contains supplementary material, which is available to authorized users.

## Background

Since the call for renewed efforts towards global malaria eradication in 2007, it has been acknowledged that new tools will be required to achieve this ambitious goal [1–5]. Drugs will need to be developed for a variety of purposes including use in elimination-focused strategies like mass drug administration (MDA) and mass screen and treat (MSAT) campaigns, prophylactic use and transmission prevention [3]. New insecticides and formulations will need to be developed considering varied vector biology and habits [5]. Importantly, diagnostic tools that are easily implemented with increased sensitivity and a decreased processing time will be necessary to quickly and successfully diagnose both patent and sub-patent infections [2]. This is particularly important as the impact of MSAT is dependent on the screening tool. South Africa is one of many countries committed to achieving malaria elimination with a target set at 2018 and all malaria-endemic provinces, including Mpumalanga province, are increasing efforts towards this ambitious goal. A malaria elimination strategy should aim to interrupt the transmission cycle and prevent it from being re-established. A successful interruption of malaria transmission ideally requires three elements: (1) the elimination of the vector, (2) the blockade of imported infections and (3) the reduction of these imported infections at their source [6]. It is unlikely that the first element will be achieved absolutely [7]. It is possible that the second element can be achieved if imported infections are identified and treated before they can contribute to the local infectious reservoir and regional collaboration is key to the success of the third element [6]. Silal et al. have simulated interventions in Mpumalanga using mathematical modelling techniques aimed towards elements (1) and (3) [8, 9]. The reduction of imported infections was dealt with at a broad level through the simulation of a focal screen and treat (FSAT) campaign at the Mpumalanga–Maputo border in Silal et al. [9]. This paper focuses in more detail on the proposed FSAT campaign at the Mpumalanga–Maputo border using a hybrid metapopulation differential equation and individual based modelling (DE-IBM) approach.

**Figure 1:**
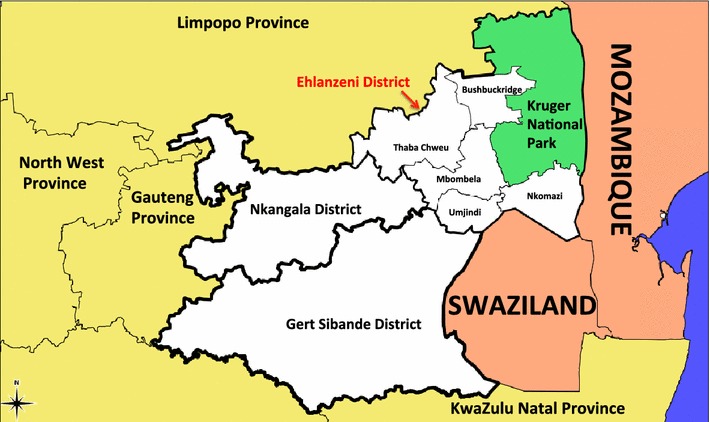
A map of Mpumalanga Province in relation to Mozambique and Swaziland [source: Mpumalanga Malaria Elimination Programme (unpublished)].

Malaria in Mpumalanga has been documented extensively [10–18]. Currently, malaria transmission occurs primarily in Ehlanzeni District on the border of both Maputo in Mozambique and Swaziland. The five municipalities in Ehlanzeni District are most affected by malaria in the province (Figure 1). The sharp decline in malaria incidence and malaria-related deaths in the province between 2002 and 2012 has been attributed to a series of policy interventions including intense vector control through indoor residual spraying (IRS), the introduction of artemisinin-based combination therapy (ACT) policy of artesunate plus sulphadoxine-pyremethamine in 2003, followed by artemether-lumefantrine (AL) in 2006 and the Lubombo Spatial Development cross-border Initiative (LSDI) between Mozambique, Swaziland and South Africa [12]. The LSDI malaria programme focused its activities primarily in Maputo Province in Mozambique and was later extended to Gaza Province resulting in substantial decreases in prevalence [19]. However the programme was terminated early in September 2010 and the resultant reduced IRS in Maputo thereafter correlates with increased malaria incidence observed from 2011 [20]. Between 2002 and 2012, 40 650 cases were notified, with the proportion of imported cases increasing from 39% in 2002 to 87% in 2012. Of the cases imported in 2012, 13% were sourced in South Africa and 85% were sourced from Mozambique (with the remaining 2% sourced from other African and Asian countries).

Compartment models and their applications in malaria in particular, have a history that spans more than 100 years [21]. Increases in computing power have led to the increased use of individual based models in recent years [22]. Metapopulation compartment models are an extension of compartment models where the population under consideration is sub-divided into patches. A compartment model is run in each patch and the patches are linked together, usually along geographical lines [23]. There have been several metapopulation compartment model applications in malaria [24–31]. There have also been several applications of IBMs in malaria [32–35]. With regards to screening and treating, Crowell et al. modelled the cost effectiveness of MSAT as a disease reduction tool in various sub-Saharan African settings [36]. MSAT was found to be effective in medium and high transmission settings and was recommended to complement and not replace interventions like active case detection and vector control. Griffin et al. [33] modelled the impact of MSAT in an African setting at various transmission intensities and found MSAT to be a complement to insecticide treated nets (ITN) usage and IRS. Maude et al. [37] modelled the impact of MSAT in the face of artemisinin resistance and found that while MSAT was able to reduce artemisinin resistant infections in the short term, it was unable to eliminate them. White et al. [38] modelled the impact of MSAT in a comparison of simple and complex mathematical models. Applications of mathematical modelling of malaria in Mpumalanga have included a climate-based fuzzy distribution model [39], an eco-hydrological model for malaria outbreaks [40] and a cluster detection model [41]. Silal et al. [8] investigated the impact of FSAT and other interventions to achieve malaria elimination in Mpumalanga using a population level compartment model and Silal et al. [9] extended this application to a metapopulation model of the five municipalities in Ehlanzeni district and Maputo province. The hybrid metapopulation DE-IBM model presented in this paper is developed to simulate the impact of FSAT at the Mpumalanga–Maputo border as a means to decrease the inflow of imported infections. This is the first model designed for this purpose in Mpumalanga and the first to do so since the call for malaria elimination in South Africa.

## Methods

### Transmission model

**Figure 2:**
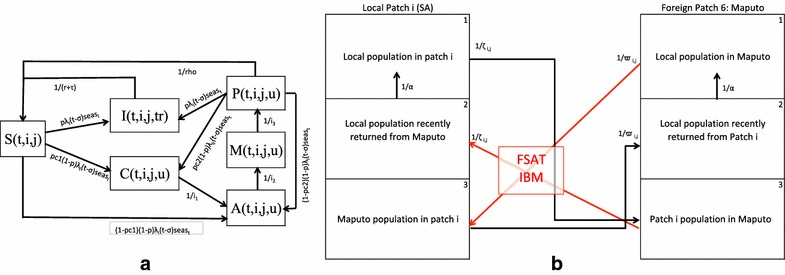
Hybrid Metapopulation DE-IBM Model flow. **a** Compartment transmission model for each patch i (*1*–*6*) with sub-patch j (*1*–*3*) at time step *t* with compartments *S* susceptible, *I* infectious and treated (tr), *C* infectious, symptomatic and untreated (u), *A* infectious, asymptomatic and untreated, *M* Infectious, sub-patent and untreated and *P* susceptible with prior asymptomatic infection. **b**) Metapopulation structure highlighting human movement between each local patch $i \ \epsilon \ \lbrace 1,2,3,4,5 \rbrace$ and foreign patch 6. Other parameters are described in Table 1 and Additional file 1.

The model presented in this paper is based on the metapopulation model described in Silal et al. [9]. The malaria transmission model has a metapopulation structure where the population of interest is divided into discrete patches under the assumption that individuals in these patches exhibit homogenous behaviour. Rather than modelling transmission in these patches in isolation, a metapopulation structure allows for transmission in a particular patch to be influenced by transmission in other patches. In this study, the area of interest is divided into six geographical patches: five patches for the five municipalities in Ehlanzeni District [Thaba Chewu (TC), Mbombela (MB), Umjindi (UJ), Nkomazi (NK) and Bushbuckridge (BB)] and one patch for Maputo province (MP). Each patch is further divided into three sub-patches representing (1) the local population currently in the patch, (2) the local population having returned from travel to a foreign place (Maputo, if the patch is South African and vice versa) and (3) the population from the foreign place currently in the patch (Figure 2b). A malaria transmission model is developed for each sub-patch where the sub-patch population is divided into six compartments representing the population susceptible to malaria (S), the population at the infectious stage that receives treatment (I), the untreated symptomatic population at the infectious stage (C), the untreated asymptomatic population at the infectious stage (A), the untreated asymptomatic, sub-patent ($<$ 100 parasites/µL) infectious population (M) and the population susceptible to malaria, but with prior asymptomatic infection (P) (Figure 2a). The liver and blood stages of the infection are incorporated as a delay in the flow between the susceptible and infectious stage compartments. Flows between compartments are governed by parameters described in Table 1. While the seasonal nature of transmission is incorporated in the model using forcing functions, the mosquito population is not modelled directly as it is assumed that the mosquito dynamics operate on a faster time-scale than the human dynamics and hence the mosquito population may be considered to be at equilibrium with respect to changes in the human population [42]. Transmission is modelled in weekly time steps.

**Table 1:** Values, descriptions and sources of the parameters driving the base metapopulation model of transmission ($i =\lbrace TC; MB; UJ; NK; BB; MP\rbrace$)

Parameter	Description	Value	Source
*N*	Population size for the six patches	$2.5 \ \times 10^6$	[58, 59]
$\mu$	Mortality/birth rate	$\frac{105}{10{,}000}$	[60]
$\sigma$	Period between liver stage and onset of gametocytemia	2 weeks	[61–64]
*r*	Artemether Lumefantrine elimination half-life	6 days	[65]
$\tau$	Time to seek treatment	1/2 weeks	Expert opinion
*ptf*	Probability of treatment failure	0.01	[51]
*p*	Proportion of local infected population receiving treatment	0.95	[66, 67]
$pf_{yr}$	Proportion of foreign infected population that receive treatment in a local patch	$pf_1 = 0.5851 \ (0.5850, 0.5853)$ (pre April 2005) $pf_2= 0.7000 \ (0.6998, 0.7010)$ (post April 2005)	Estimated from model fitting process
$i_1$	Duration of clinical infection before becoming asymptomatic	0.7 weeks	[33]
$i_2$	Duration of asymptomatic infection before becoming sub-patent	5.5 weeks	[33, 68]
$i_3$	Duration of sub-patent infection	24 weeks	[33]
$\rho$	Duration of clinical immunity	5 years	[69]
$pc_1$	Probability of clinical infection from naive individuals	0. 9997 (0.9756, 0.9999)	[63, 70]
$pc_2$	Probability of clinical infection from partially immune individuals	0.883 (0.877, 0.888)	Estimated from data
$seas_i$	Seasonal forcing function for foreign sourced cases	Derived from data	[10]
$\beta _i$	Annual number of mosquito bites per person × proportion of bites testing positive for sporozoites for patch *i*	$\beta _{TC} = 4.488 \ (4.178, 4.798)$ $\beta _{MB} = 6.034 \ (5.967, 6.101)$ $\beta _{UJ} = 0.655 \ (0.589, 0.723)$ $\beta _{NK} = 1.546 \ (1.521, 1.571)$ $\beta _{BB} = 4.436 \ (4.264, 4.609)$ $\beta _{MP} = 99.065 \ (98.920, 99.210)$	Estimated from model fitting process
$\lambda _i(t)$	Force of infection	See Additional file 1	
$\frac{1}{\alpha }$	Rate of assimilation of population in sub-patch 2 (locals having returned from foreign travel) back into sub-patch 1 from whence they originated	1.5 week^−1^	Expert opinion
$\frac{1}{k}$	Rate of movement between five Mpumalanga municipalities	1/ 201.436 (1/204.833, 1/198.040) week^−1^	Estimated from model fitting process
$\frac{1}{v_{yr}}$	Maputo residents: rate of movement between Maputo and five Mpumalanga municipalities	$\frac{1}{v_1}= 1/7{,}616.743$ week^−1^ $(1/7{,}663.186, 1/7{,}570.299)$ (pre April 2005) $\frac{1}{v_2}= 1/3{,}227.213$ week^−1^ $(1/3{,}187.684, 1/3{,}266.742)$ (post April 2005)	Estimated from model fitting process
$\frac{1}{\varpi _{i,j}}$	Maputo residents: rate of movement between Maputo and 5 Mpumalanga municipalities based on $\frac{1}{v_{yr}}$ and distance between patches	See Additional file 1	
$\frac{1}{z}$	Mpumalanga residents: rate of movement between 5 Mpumalanga municipalities and Maputo	$\frac{1}{z}= 1/359.462$ week^−1^ (1/361.057, 1/357.866)	Estimated from model fitting process
$\frac{1}{\zeta _{i,j}}$	Mpumalanga residents: rate of movement between 5 Mpumalanga municipalities and Maputo based on $\frac{1}{z}$ and distance between patches	See Additional file 1	
*fwgt*	Foreign movement weight intensity	10.615 (10.512, 10.719)	Estimated from model fitting process
*lwgt*	Local movement weight intensity	1.419 (1.343, 1.495)	Estimated from model fitting process
*vef*	Effectiveness of vector control	0.9785 (0.9783, 0.9787)	Estimated from model fitting process
$vc_i[t]$	Vector control coverage in patch *i* $\times$ efficiency	Derived from data	

Human movements between patches are modelled in two ways. Local travel may occur between the five Mpumalanga patches (from all five compartments in all three sub-patches). Foreign travel may occur between the Maputo patch and the five Mpumalanga patches (from all five compartments) as illustrated in Figure 2b. These movements are inversely weighted by distance so that movements between patches that are closer together are more likely than movements between patches that are further apart. A full description of the metapopulation transmission model is presented in Additional file 1.

### FSAT model

**Figure 3:**
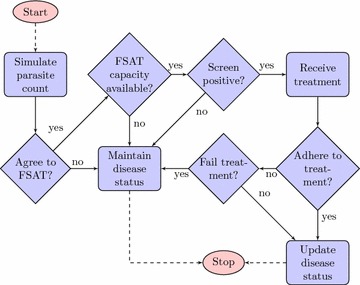
FSAT IBM algorithm.

Figure 2b shows that only movement of the local Mpumalanga population returning from Maputo (patch 6, sub-patch 3 to patches 1–5, sub-patch 2) and the foreign population travelling to Mpumalanga from Maputo (patch 6, sub-patch 1 to patches 1–5, sub-patch 3) are subject to the FSAT campaign as the purpose of the campaign is to prevent infections from entering Mpumalanga. The FSAT model has an individual-based model structure so that individual characteristics of the participants may be taken into account. Figure 3 depicts the algorithm applied to individuals in the FSAT model. The flow of local and foreign populations from Maputo into Mpumalanga at each time step (week) is captured and geographical destination patch, the sub-patch and disease status (susceptible, infectious, sub-patent etc) are stored for each individual in that flow. The first step is to simulate a parasite count for each individual dependent on their disease status. The log-normal distribution was selected with distribution parameters in Table 2 as it captures the skewness of parasite count distribution. To test the impact of take-up proportion, the campaign is modelled as being optional. Should an individual not wish to be part of the campaign, their disease status is maintained and the simulation is stopped. Depending on the diagnostic tool used, the processing times and hence the number of tests able to be performed per week will differ. Should capacity be available and an individual agrees to participate in the campaign, the individual is screened. A positive screen occurs if the individual’s simulated parasite load is greater than the detection threshold of the diagnostic tool in use. A positive screen will result in the individual being treated. As the treatment is likely to be a multiple-dose regimen, there is a chance that the individual may not adhere to the full course and may run the risk of failing treatment. In the event of successful treatment, the individual’s disease status is updated (e.g. Infectious to “Infectious having received FSAT” where the individual is cured from malaria at a rate of recovery dependent on the parasite clearance time of the drug) and the simulation stops. The model parameters governing this IBM algorithm are displayed in Table 2.

**Table 2:** Values, descriptions and sources of the parameters driving the FSAT Individual Based Model

Parameter	Description	Value	Source
*fson*	Focal Screen and Treat Switch	Binary	
*cov*	FSAT coverage	25; 50; 75; 100%	Values to be tested
	Baseline FSAT coverage	70%	Assumed
*fsprop*[*t*]	Proportion Screened and Treated through Border Control	fson $\times$ cov	
*opt*	Take-up proportion for FSAT	25; 50; 75; 100%	Values to be tested
*adh*	Probability of adherance	0.90	[51]
*fail*	Probability of treatment failure	0.01	[51]
*rep*	Number of screens tests performed simultaneously	3	Assumed
$\mu _C$	Geometric mean of log-normal parasite distribution for clinical infections	25,000	[71, 72]
$\sigma _C$	Log standard deviation of log-normal parasite distribution for clinical infections	1.3	[71, 72]
$\mu _A$	Geometric mean of log-normal parasite distribution for asymptomatic infections	1,000	[71, 73]
$\sigma _A$	Log standard deviation of log-normal parasite distribution for asymptomatic infections	1.5	[71, 73]
$\mu _S$	Geometric mean of log-normal parasite distribution for sub-patent infections	50	[55]
$\sigma _S$	Log standard deviation of log-normal parasite distribution for sub-patent infections	0.75	Assumed

### Hybrid metapopulation DE-IBM model

The metapopulation DE model and the IBM model are linked such that the IBM model is nested in the DE model. At each time step, the DE model generates flows of a population that leave one compartment and enter another compartment (in the various sub-patches and patches). The IBM model takes the flow value at each time step once it has been negated from a compartment, discretises it into individuals in a population, executes the IBM algorithm, re-groups the individuals back into a population flow, and adds the flow to its destination compartment. In this application, only the flows of local and foreign people entering the five Mpumalanga patches from Maputo are interrupted to perform FSAT using the IBM model. Further details on this hybrid modelling approach are available in the Additional file 1.

### Data fitting

The metapopulation transmission model is fitted to weekly case notification data from Mpumalanga and Maputo Province from 2002 to 2008, and then validated with data from 2009 to 2012. Ethical approval for use of this secondary data was obtained from the Mpumalanga Department of Health and the University of Cape Town Human Research Ethics Committee. The Mpumalanga case data displays a characteristic triple peaked pattern in the malaria season with peaks occurring in September/October, December/January and April/May while the Maputo Province malaria season exhibited peaks in December and April only [10]. The seasonal forcing functions, used to determine seasonal variation in transmission, for the six patches are derived from the data using seasonal decomposition of time series by LOESS (STL) methods for extracting time series components [43]. ACT drug therapy and the impact of IRS implemented between 2002 and 2008 are also included in the model. In order to reach a steady state the model is run deterministically from 1990 before being fitted to data from 2002. The model output (predicted weekly treated cases) is fitted to the data from 2002 to 2008 using the maximum likelihood approach by assuming an underlying Poisson distribution with rate $\lambda$ as the number of treated cases per week. Several parameters as detailed in Table 1 are estimated through the data fitting process using the population-based global search algorithm of particle swarm optimisation [44, 45]. The model with the estimated parameter values is then validated with a further 3 years of data (2009–2012). A full description of the data fitting method is presented in Additional file 1. All model development, fitting and subsequent analysis was performed in R v3.02 [46]. The particle swarm optimisation routine was performed using the R package hydroPSO v0.3-3 [47, 48].

### Simulated FSAT

An FSAT campaign is tested on a stochastic version of the fitted model; the same intervention is applied to multiple model runs such that its impact on local infections can be described with a mean effect and a 95% confidence interval. Stochastic uncertainty and parameter sensitivity has been accounted for as follows. The model is run stochastically by treating each flow between compartments at each time point *t* as a random realisation of a Poisson process with rate $\lambda$, the deterministic flow value at that time, and by simulating the parameter values uniformly from their 95% confidence intervals. The predicted impact of an FSAT campaign at the Mpumalanga–Maputo border is presented with respect to coverage levels, thresholds of detection, take-up proportions, target levels and typical diagnostic tools. To facilitate accurate comparison of coverage levels, targets, thresholds and diagnostic tools, the take-up proportion of FSAT is fixed at 100%. Take-up proportion itself is explored at low, intermediate and high levels. The FSAT campaign is assumed to run for 8 h a day, seven days a week with a maximum of three tests being conducted simultaneously. This number of simultaneous tests is also considered at different levels in the simulation. As malaria elimination is defined by the World Health Organisation as zero incidence of locally contracted cases, the impact of the simulated FSAT campaign is measured as the decrease in local infections as result of the campaign [7]. This impact is a function of the change in onward transmission resulting from fewer imported infections entering Mpumalanga (due to FSAT). All results are compared to the base case of no FSAT, depicted in black in all figures. Each scenario was run 450 times so that results presented are the mean local infections per week with a 95% confidence interval shaded around the mean. In many cases, the shading is not visible due to either narrow confidence intervals or a low resolution y-axis.

### Diagnostic tools

Diagnosing malaria at a border point ideally requires a diagnostic tool that is both sensitive, specific and has a short processing time. Several tools have been considered for this simulation (Table 3). The Rapid Diagnostic Test (RDT) currently in use at South African public health facilities has a theoretical detection threshold of 200 parasites/µL and a maximum processing time of 20 min. Microscopy in experienced hands may exhibit a sensitivity of 50 parasites/µL but is more likely to have a sensitivity in the region of 100 parasites/µL. Real-time quantitative polymerase chain reaction (qPCR) and loop-mediated isothermal DNA amplification (LAMP) are very sensitive tools with qPCR needing sophisticated equipment for a processing time of 3 h. LAMP on the other hand is a less complex technique with a 1 h processing time [49]. These diagnostic tools are also compared to a highly sensitive hypothetical RDT with a standard process time of 20 min and a detection threshold of 5 parasites/µL.

**Table 3:** Descriptions of diagnostic tools used in FSAT model

Tool	Detection threshold (parasites/µL)	Process time (h)	Target per week (tests per/h $\times$ 3 reps $\times$ 8 h $\times$ 7 days)	Source
RDT	200	0.33	504	[49]
Microscopy	100	2.25	75	Expert opinion, [49]
qPCR	1	3	63	[49]
LAMP	5	1	168	[74, 75]
Hypothetical RDT	5	0.33	504	

## Results

The estimation of parameters through data fitting is presented first followed by the results of a simulated FSAT campaign at varied coverage levels, target levels and thresholds of detection.

### Estimation of parameters through data-fitting

**Figure 4:**
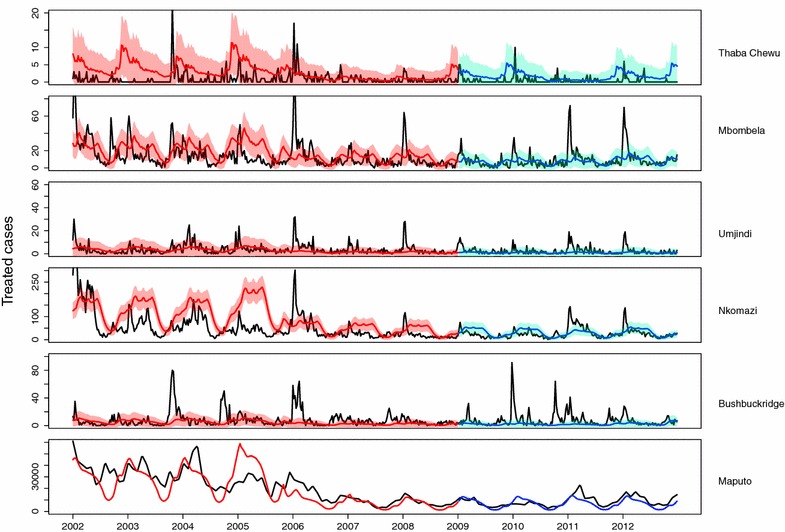
Predicted weekly treated cases (*blue* 2002–2008; *red* 2009–2012) fitted to and validated with data (*black*). The 95% uncertainty range for weekly case predictions is shown.

Weekly case data for the five Mpumalanga patches and Maputo (black) along with the model output from the data-fitting process (red) and predicted model output for 2009 to 2012 (blue) is shown in Figure 4. Figure 4 is a summation of the data fitting results in each patch, as data was fitted to each sub-patch simultaneously. Both the peak and timing of the malaria season in Ehlanzeni District and Maputo Province are captured well by the model prediction and the uncertainty range. Additional file 1 contains more detailed output from the data-fitting procedure. The parameters estimated through data-fitting and other parameters driving the transmission model are presented in Table 1. There are two rates each for the foreign treatment proportion and the rate of foreign movement. This is due to the waiver of short stay visa requirements between Mozambique and South Africa in April 2005, resulting in increased cross-border movement [50].

#### Diagnostic tools

**Figure 5:**
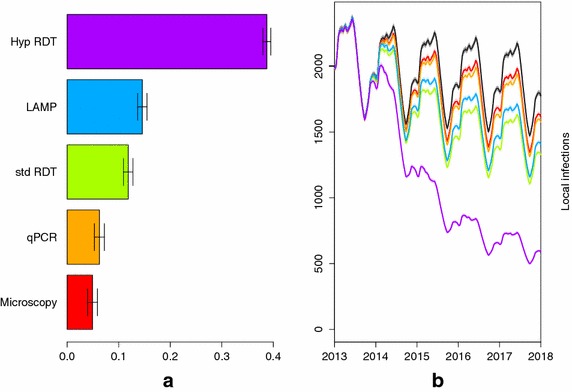
Predicted impact due to FSAT between 2014 and 2018 using the following diagnostic tools: microscopy (*red*), qPCR (*orange*), RDT (*green*), LAMP (*blue*) and a hypothetical RDT (*purple*). **a** Shows the percentage decrease in local infections due to the FSAT and **b** shows the impact of FSAT on local infections in Ehlanzeni district through time compared to the base case of no interventions (*black*).

The model predicts that at a baseline FSAT coverage of 70%, microscopy and qPCR do not substantially decrease local infections due to their long processing time (Figure 5). LAMP and the RDT with a detection threshold of 200 parasites/µL are predicted to lead to larger decreases in local infections. LAMP is more likely to detect asymptomatic and sub-patent infections than the RDT, but the three times longer turnaround time results in only a third of the tests being performed compared to the RDT. The hypothetical RDT that is as sensitive as LAMP is predicted to perform the best as it combines a high sensitivity with a short processing time.

#### Coverage, detection thresholds, take-up proportions and target levels

**Figure 6:**
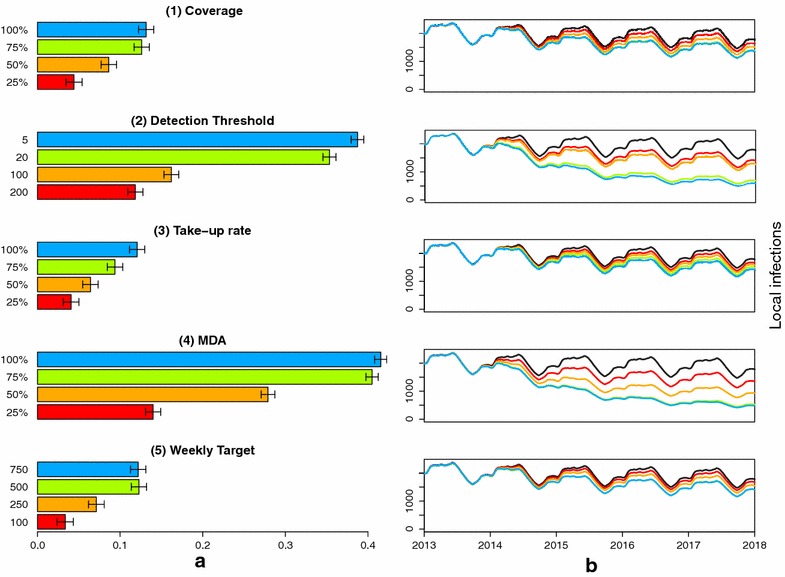
Predicted impact due to FSAT between 2014 and 2018. **a** Shows the percentage decrease in local infections due to the FSAT and **b** shows the impact of FSAT on local infections in Ehlanzeni district through time compared to the base case of no interventions (*black*). The impact of FSAT is predicted for different (*1*) coverage proportions, (*2*) thresholds of detections for the diagnostic tool used (parasites/µL), (*3*) take-up proportions, (*4*) coverage proportions for mass drug administration and (*5*) weekly targets (capacity) keeping all other variables constant. 95% confidence intervals are depicted for the average percentage decrease. The *colours of the bars* in (**a**) correspond to the level of local infections depicted in (**b**).

Coverage levels refer to the proportion of cross-border movements reached by the FSAT campaign. This proportion is generally less than 100% to account for other forms of entry into the province e.g. illegal entry. FSAT at various coverage levels is simulated using the characteristics of the RDT currently in use in South Africa (theoretical detection threshold of 200 parasites/µL and a process time of 20 min. Figure 6(1) shows that even at 25% coverage, a substantial decrease in local infections is predicted. The marginal impact on local infections decreases to the point where local infections at 75 and 100% FSAT coverage are non-distinguishable with overlapping confidence intervals for the expected decrease in local infections. This occurs because though coverage may be very high, infections with less than 200 parasites/µL will remain undetected by a standard RDT.

In simulating the impact of detection thresholds alone, coverage is kept at its baseline value of 70%. Figure 6(2) shows that the predicted impact on local infections of using an RDT at any threshold is substantial. The greatest decrease in local infections is predicted with the most sensitive RDT. The impact of take-up proportion is such that a low proportion of only 25% does not substantially decrease local infections compared to the extreme of 100% take-up i.e. mandatory participation (Figure 6(3)). The local infections predicted by mandatory participation may be further decreased if a more sensitive tool was used, or coverage was higher. If mandatory participation is a viable option, a government could consider a mass drug administration instead of FSAT. Figure 6(4) suggests that at even 50% coverage, the impact of MDA is higher than FSAT at 100% coverage as sub-patent infections are being captured by the intervention. A baseline assumption is that three screenings may be performed simultaneously. If this capacity is increased, the number of tests that may be performed per week will also increase. Comparing different weekly target levels suggests that any target below 250 people is not predicted to substantially decrease local infections (Figure 6(5)).

A sensitivity analysis was conducted to assess the effect of varying coverage, detection thresholds, take-up proportions, adherence and target levels *simultaneously* in addition to the one-at-a-time analysis conducted above. The decrease in local infections was measured for each combination of factors and a linear model regressing these five factors on the decrease in local infections was fitted to assess sensitivity. The standardised regression coefficients in Table 4 suggest that holding the other factors constant, detection threshold in an FSAT campaign has the largest absolute impact on decreasing local infections, followed by coverage achieved, take-up proportion and target level. This is in line with Figures 5 and 6 where the largest decreases were achieved for the most sensitive diagnostic tool. Adherence was the only factor to have a non-significant impact on the decrease in local infections, primarily because the probability of treatment failure assumed in this study is only 0.01 [51]. With the rise of artemisinin resistance, it is likely that the probability of treatment failure will increase, and adherence will be of greater importance than this simulation study has shown.

**Table 4:** Sensitivity analysis of factors assessed in FSAT model

Factor	Standardised regression coefficient	95% confidence interval
Coverage	0.41795	(0.38181, 0.45409)
Take-up proportion	0.36715	(0.33100, 0.40329)
Adherence	0.00095	(−0.03520, 0.03709)
Detection threshold	−0.47861	(−0.51475, −0.44247)
Target level	0.34027	(0.30413, 0.37642)

## Discussion

This paper presented a hybrid DE-IBM model where a metapopulation level, non-linear stochastic ordinary differential equation model was used to simulate malaria transmission in Mpumalanga and Maputo so that Individual-Based Modelling techniques may be used to predict the impact of FSAT at the Mpumalanga–Maputo border. The model has predicted that the various scenarios of FSAT considered in this paper will decrease the number of local infections, but not eliminate malaria. Testing FSAT at various levels of coverage, take-up, detection thresholds and targets suggested that decreases in local infections are most sensitive to the detection threshold, and hence the diagnostic tool used, followed by coverage achieved. Von Seidlein [52] lists both the inability of FSAT to detect and treat all infections (using RDTs), and hence the inability to prevent reinfection as reasons why screening and treating failed in trials conducted at a school level [53]. This study and those discussed earlier suggest that as long as the reservoir of sub-patent infections endures, FSAT on its own will not be able to eliminate malaria. The model has predicted through various scenarios of FSAT, that it may still be successful in *reducing* local malaria incidence, even if it cannot reduce it to zero. In this manner, FSAT may still form part of an integrated elimination strategy where a variety of interventions are employed together to achieve malaria elimination.

In the last decade, a policy shift to ACT, source reduction through vector control in Mozambique in the LSDI malaria programme and a strong IRS strategy have contributed to decreasing malaria prevalence in Mpumalanga to the low levels experienced today. In shifting focus from control to elimination, the goal to interrupt transmission and prevent its reestablishment implies that a “more of the same” approach will not work [6]. The early termination of the LSDI programme and the associated subsequent rise in cases in Maputo Province and Mpumalanga demonstrate the importance of regional collaboration and urgency to collaborate further. Silal et al. assessed the impact of source reduction in Maputo on malaria incidence in Mpumalanga using a metapopulation model of transmission [9]. That model predicted that the largest decrease in local infections was achieved when source-reducing interventions were simulated. Of the three ideal elements of an elimination strategy highlighted in the introduction of this paper, the prevention of imported infections is not addressed by a strong IRS focus as IRS will limit onward transmission of all infections but is not targeted at imported infections. The implementation of FSAT at the border is one strategy to inhibit the inflow of local and foreign individuals with malaria infections sourced elsewhere. Successful implementation of this strategy requires among other things, decisions on when to conduct the campaign, the choice of diagnostic tool and drug and the level of resources and man-power available. Silal et al. used a population level transmission model for Mpumalanga to show that FSAT conducted at the Mpumalanga–Maputo border over the peak of the season only is not as effective in decreasing local infections because imported infections resume previous high levels as soon as FSAT is stopped [9]. Sustained decreases were predicted when FSAT was conducted at the border throughout the year.

The choice of diagnostic tool is a critical one for several reasons. Firstly, the tool should be highly sensitive to effectively screen and treat all infections. If a tool with a low to medium sensitivity is used, it is likely that sub-patent infections will be missed by FSAT and enter Mpumalanga to contribute to infectious reservoir and thus to onward transmission. Okell et al. estimated that in very low prevalence settings, sub-patent infections comprise 70–80% of all malaria infections and are responsible for 20–50% of all human-to-mosquito infections [54]. Without addressing these sub-patent infections, it is likely that these low density infections will sustain malaria transmission [55]. Secondly, the diagnostic tool should take into account existing man-power and resources. Some tools such as qPCR require specialised equipment and highly trained operators while other tools like RDTs and LAMP have protocols that require minimal instrumentation and expertise [49]. Thirdly, the processing time of the tools will directly impact the usefulness of the tool in FSAT. Tools with long turnaround times are not feasible as it is unlikely that travellers would be willing to wait at a border-post for a result that takes a long time to process. In simulating the choice of diagnostic tool, the model predicted that microscopy and qPCR, though more sensitive than RDTs performed worse than LAMP and RDTs owing to the long processing times. LAMP may be an ideal tool for FSAT when the population of interest is able to wait for an hour to receive results, but this is most likely not the case for individuals passing through a border-post. The best performing tool was predicted to be a hypothetical RDT that had the standard processing time of 20 min with the sensitivity of LAMP. Modelling this hypothetical tool resulting in a capacity of 504 tests per week. The same target would be reached if increased resources resulted in nine LAMP tests being performed simultaneously instead of the baseline three tests.

From a provider perspective, the success of a FSAT strategy depends critically on the allocated capital and labour resources. Resources will influence the length of the FSAT campaign, the choice of tool and the labour assigned to the execution of the campaign. The model has predicted that processing a small proportion of individuals passing through the border results in small decreases in local infection. To optimize the impact of a FSAT campaign, the choice of tool and labour assigned to implement the tool should together seek to maximise the number of tests possible. From a participant perspective, the success of a FSAT campaign depends on the willingness to participate in the campaign and after a positive test result, the adherence of the participant to complete the drug regimen. The first line of malaria treatment in South Africa is a three day regimen of artemether-lumefantrine where only the first dose is supervised [56]. The issue of drug adherence may only be addressed adequately when a single dose cure of malaria is available. The model predicts that a high willingness to participate results in substantially larger decreases in local infections. One method to guarantee participation is to make the FSAT campaign mandatory. This approach would most likely require extensive resources to cope with the workload and enable efficient passing through the border, but may also be against the ethos of the South African and Mozambican governments. Yet even with mandatory FSAT, sub-patent infections may be missed depending on the screening tool employed. On the other hand, the model predicted that MDA at a low coverage in place of FSAT leads to large decreases in local infections as low sensitivity of screening tools is no longer an issue. Given recent estimates that 70–80% of all infections in a low transmission setting are sub-patent, MDA at the border becomes a suitable intervention [54]. An alternative way to view MDA at border entry points, is mandatory prophylaxis for travellers. Many countries require proof of vaccinations against yellow fever, typhoid, influenza and other diseases and infections. Proof of recent malaria prophylaxis can then effectively become a “lower cost” mass drug administration campaign for all travellers (local and foreign) travelling from a malaria-endemic country. If this becomes policy, the MDA campaign is effectively extended to all ports of entry into a country, not just the few selected for a traditional MDA/FSAT campaign. A 2003 study on Travellers’ Knowledge, Attitudes and Practices at Johannesburg’s O.R. Tambo International Airport revealed that 74% of respondents to the malaria questionnaire were carrying antimalarials in the form of prophylaxis or standby emergency treatment [57].

This is the first detailed FSAT study in South Africa and Mpumalanga. A lack of data on border crossings has resulted in the need to estimate migration rates from the transmission data and perform a sensitivity analysis on the results (Additional file 1). Future work includes migration data as it becomes available, extending the model to Southern Africa and adding an economic cost component to the FSAT IBM model in an attempt to optimize the impact on local infections based on a suite of potential diagnostic tools and the resources required to implement them.

## Conclusion

Malaria incidence and related mortality has declined since 2002 to a point where South Africa is in the pre-elimination phase ($<$5 cases per 1,000). In this time the proportion of annual imported cases has increased from 39% in 2002 to 87% in 2012. The reduction of imported cases will be vital to any future malaria control or elimination strategy. Mathematical modelling has been used in this paper to estimate the impact of FSAT at a border control point under a variety of scenarios, as a means to reduce the inflow of imported infections in an environment where imported cases far exceed local cases. In this manner, mathematical modelling of FSAT may be used to inform a strategy with a strong regional focus aimed at interrupting local transmission so that malaria elimination may one day become a reality.

## Additional file

Additional file 1:
**Mathematical model description.** Additional file contains the model description, model equations and additional information on the data-fitting process.
