# Supplementary material for: Predicting the impact of border control on malaria transmission: a simulated focal screen and treat campaign
Source: Malar J. 2015 Jul 12;14:268. doi: 10.1186/s12936-015-0776-2 (PMC4499227; doi:10.1186/s12936-015-0776-2)
Supplement: Additional file 1: — Mathematical model description. Additional file contains the model description, model equations and additional information on the data-fitting process. [file 12936_2015_776_MOESM1_ESM.pdf]

Predicting the impact of border control on malaria transmission: a simulated focal screen and treat campaign

Additional File 1: Mathematical Model Description

Sheetal P Silal *[1], Francesca Little [1] , Karen I Barnes [2] and Lisa J White [3,4]

[1]Department of Statistical Sciences, University of Cape Town, Cape Town, South Africa
[2]Division of Clinical Pharmacology, Department of Medicine, University of Cape Town, Cape Town, South Africa
[3]Mahidol-Oxford Tropical Medicine Research Unit, Mahidol University, Bangkok, Thailand
[4]Centre for Tropical Medicine, Nuffield Department of Clinical Medicine, Churchill Hospital, University of Oxford, Oxford, UK

Email: Sheetal P Silal *- sheetal.silal@uct.ac.za; Francesca Little - francesca.little@uct.ac.za; Karen I Barnes - karen.barnes@uct.ac.za; Lisa J White - lisa@tropmedres.ac;

*Corresponding author

Summary Equations: Metapopulation Model of Transmission

The model presented in this paper is based on a model described in Silal *et al.* [1]. The model comprises six patches; five to represent Mpumalanga municipalities, and one for Maputo province, Mozambique. Each patch comprises three sub-patches: (1) the local population of patch i currently in patch i, (2) the local population of patch i having returned from travel to a foreign place (Maputo, if the patch is South African and vice versa) and (3) the population from the foreign place currently in patch i. A malaria transmission model is developed for each sub-patch where the sub-patch population is divided into six compartments representing the population susceptible to malaria (S), the population at the infectious stage that receives treatment (I), the untreated symptomatic population at the infectious stage (C), the untreated asymptomatic population at the infectious stage (A), the untreated asymptomatic, sub-patent (< 100 parasites/μs) infectious population (M) and the population susceptible to malaria, but with prior asymptomatic infection (P). The liver and blood stage of the infection is incorporated as a delay in the flow between the susceptible and infectious stage compartments.

Descriptions of the compartments and parameters governing the flows between compartments are provided inTables 1 and 2 respectively. This model structure differs from that presented in Silal *et al.* in two ways [1]. Firstly, given that the purpose of this paper is to predict the impact of FSAT, it is necessary to

model infectious stage infections in more detail. The sensitivity of diagnostic screening tools is such that sub-patent infections, i.e. infections with a low parasite count, may often be missed. These infections then remain untreated and continue to contribute to the infectious reservoir in the area. For this reason, the untreated infectious stage compartment in Silal *et al* is decomposed into the C, A and M compartments described earlier. Secondly, the blood stage of infection is incorporated as a delay in the flow between the susceptible and infectious stage compartments in order to simplify the model structure given the addition of asymptomatic and sub-patent infection compartments.

The force of infection $\lambda_i[t]$ for each patch i is a function of the level of vector control, the annual number of mosquito bites per person x proportion of bites testing positive for sporozoites for patch i (β_i) and the proportion of infectiousness in the population lagged to reflect the infectiousness proportion at the time the mosquito was infected.

$$\lambda_i[t] = (1 - vc_i[t] * vef)\beta_i \times \frac{\sum_{k=1}^{3}(I_{i,k}[t-4] + C_{i,k}[t-4] + A_{i,k}[t-4] + M_{i,k}[t-4])}{N_i[t-4]}.$$

Population migration between the different patches is characterised by three sets of movements:

1. Movement may occur between any two of the the five Mpumalanga patches i and j at a rate $\frac{1}{\kappa_{i,j}}$ where

$$\frac{1}{\kappa_{i,j}} = \frac{1}{k} \times \frac{\frac{1}{(1+\sqrt{(x_i-x_j)^2+(y_i-y_j)^2})^{lwgt}}}{\sum_{i=1}^{5}\frac{1}{(1+\sqrt{(x_i-x_j)^2+(y_i-y_j)^2})^{lwgt}}} \qquad \text{if } i,j = 1,2,3,4,5 \ \& \ i \neq j$$

$$= 0 \qquad \text{if } i \text{ or } j = 6 \text{ (local movement only)}$$

$$\frac{1}{\kappa_{i,j}} = \frac{1}{\kappa_{j,i}}$$

where (x_i, y_i) and (x_j, y_j) are the centroid coordinates for patches i and j respectively. This movement is weighted inversely by distance so that movement between South African patches that are closer together occurs at a higher rate than those further apart.

2. Movement may occur when South African citizens cross the border into Maputo (from patch $i = 1-5$ in sub-patch 1 to patch 6 in sub-patch 3) and return (patch 6 in sub-patch 3 to patch $i = 1-5$ in sub-patch 2) at a rate of $\frac{1}{\zeta_{i,6}}$ where

$$
\begin{aligned}
\frac{1}{\zeta_{i,6}} &= \frac{1}{z} \times \frac{\frac{1}{(1+\sqrt{(x_i-x_6)^2+(y_i-y_6)^2})^{fwgt}}}{\sum_{i=1}^{5} \frac{1}{(1+\sqrt{(x_i-x_6)^2+(y_i-y_6)^2})^{fwgt}}} \qquad \text{if } i = 1,2,3,4,5 \\
&= 0 \qquad \text{otherwise} \\
\frac{1}{\zeta_{i,6}} &= \frac{1}{\zeta_{6,i}}
\end{aligned}
$$

where (x_i, y_i) and (x_6, y_6) are the centroid coordinates for patches i and 6 respectively. This movement is weighted inversely by distance so that movement between the South African patches and Maputo that are closer together occurs at a higher rate than those further apart.

3. Movement may also occur when Mozambican citizens cross the border into Mpumalanga (from patch 6 in sub-patch 1 to patch $j = 1-5$ in sub-patch 3) and return (patch $j = 1-5$ in sub-patch 3 to patch 6 in sub-patch 2) at a rate of $\frac{1}{\varpi_{6,j}}$ where

$$
\begin{aligned}
\frac{1}{\varpi_{6,j}} &= \frac{1}{\upsilon_{yr}} \times \frac{\frac{1}{(1+\sqrt{(x_6-x_j)^2+(y_6-y_j)^2})^{fwgt}}}{\sum_{i=1}^{5} \frac{1}{(1+\sqrt{(x_6-x_j)^2+(y_6-y_j)^2})^{fwgt}}} \qquad \text{if } j = 1,2,3,4,5 \ \&\ yr = 1,2 \\
&= 0 \qquad \text{otherwise} \\
\frac{1}{\varpi_{6,j}} &= \frac{1}{\varpi_{j,6}}
\end{aligned}
$$

where (x_6, y_6) and (x_j, y_j) are the centroid coordinates for patches 6 and j respectively. This movement is weighted inversely by distance so that movement between the South African patches and Maputo that are closer together occurs at a higher rate than those further apart.

This leads to the following set of differential equations.

Sub-patch 1 (Local population): For each patch i with population movement (local or foreign) to patch j $(i, j\ \epsilon\{1,2,..,6\}, j \neq i\ )$:

$$
\frac{dS_{i,1}}{dt} = \underbrace{\mu N_i}_{(1)} - \underbrace{\lambda_i[t-\sigma]seas_i[t]S_{i,1}}_{(2)} + \underbrace{\frac{1}{r+\tau}I_{i,1}}_{(3)} + \underbrace{\frac{1}{\rho}P_{i,1}}_{(4)} + \underbrace{\frac{1}{\alpha}S_{i,2}}_{(5)} + \underbrace{\sum_j \frac{1}{\kappa_{i,j}}(S_{j,1} - S_{i,1})}_{(6)} - \underbrace{\frac{1}{\varpi_{i,j}}S_{i,1}}_{(7)} - \underbrace{\frac{1}{\zeta_{i,6}}S_{i,1}}_{(8)} - \underbrace{\mu S_{i,1}}_{(9)}
$$

$$
\frac{dI_{i,1}}{dt} = \underbrace{p\lambda_i[t-\sigma]seas_i[t](S_{i,1}+S_{i,2})}_{(10)} + \underbrace{p\lambda_i[t-\sigma]seas_i[t](P_{i,1}+P_{i,2})}_{(11)} - \frac{1}{r+\tau}I_{i,1} + \frac{1}{\alpha}I_{i,2} + \sum_j \frac{1}{\kappa_{i,j}}(I_{j,1} - I_{i,1})
$$

$$
\begin{aligned}
&- \frac{1}{\varpi_{i,j}} I_{i,1} - \frac{1}{\zeta_{i,6}} I_{i,1} - \mu I_{i,1} \\
\frac{dC_{i,1}}{dt} &= \underbrace{pc_1(1-p)\lambda_i[t-\sigma]seas_i[t](S_{i,1}+S_{i,2})}_{(12)} + \underbrace{pc_2(1-p)\lambda_i[t-\sigma]seas_i[t](P_{i,1}+P_{i,2})}_{(13)} - \underbrace{\frac{1}{i_1} C_{i,1}}_{(14)} + \frac{1}{\alpha} C_{i,2} + \\
&\sum_j \frac{1}{\kappa_{i,j}} (C_{j,1} - C_{i,1}) - \frac{1}{\varpi_{i,j}} C_{i,1} - \frac{1}{\zeta_{i,6}} C_{i,1} - \mu C_{i,1} \\
\frac{dA_{i,1}}{dt} &= \underbrace{(1-pc_1)(1-p)\lambda_i[t-\sigma]seas_i[t](S_{i,1}+S_{i,2})}_{(15)} + \underbrace{(1-pc_2)(1-p)\lambda_i[t-\sigma]seas_i[t](P_{i,1}+P_{i,2})}_{(16)} + \frac{1}{i_1} C_{i,1} \\
&- \underbrace{\frac{1}{i_2} A_{i,1}}_{(17)} + \frac{1}{\alpha} A_{i,2} + \sum_j \frac{1}{\kappa_{i,j}} (A_{j,1} - A_{i,1}) - \frac{1}{\varpi_{i,j}} A_{i,1} - \frac{1}{\zeta_{i,6}} A_{i,1} - \mu A_{i,1} \\
\frac{dM_{i,1}}{dt} &= \frac{1}{i_2} A_{i,1} - \frac{1}{i_3} M_{i,1} + \frac{1}{\alpha} M_{i,2} + \sum_j \frac{1}{\kappa_{i,j}} (M_{j,1} - M_{i,1}) - \frac{1}{\varpi_{i,j}} M_{i,1} - \frac{1}{\zeta_{i,6}} M_{i,1} - \mu M_{i,1} \\
\frac{dP_{i,1}}{dt} &= \underbrace{\frac{1}{i_3} M_{i,1}}_{(18)} - \lambda_i[t-\sigma]seas_i[t](P_{i,1}+P_{i,2}) - \frac{1}{\rho} P_{i,1} + \frac{1}{\alpha} P_{i,2} + \sum_j \frac{1}{\kappa_{i,j}} (P_{j,1} - P_{i,1}) - \frac{1}{\varpi_{i,j}} P_{i,1} \\
&- \frac{1}{\zeta_{i,6}} P_{i,1} - \mu P_{i,1}
\end{aligned}
$$

(1) Births in patch i

(2) Local incidence arising from sub-patch 1

(3) Recovery of treated infectious stage infections at a rate dependent on the time to seek treatment and the time to recovery

(4) Return to full susceptibility at a rate determined by the duration of clinical immunity

(5) Assimilation of population in sub-patch 2 (locals having returned from foreign travel) back into sub-patch 1 from whence they originated.

(6) Movement between local patches (1-5) out of and into the compartment

(7) Movement of local patch i population to foreign patch j when i=6 and j = 1-5; =0 for all other values of i as this rate is particular to movement of Maputo population (patch 6)

(8) Movement of local patch i population to foreign patch 6 when i=1-5; =0 for i=6 as this rate is particular to movement of the Mpumalanga population to and from Maputo (patches 1-5)

(9) Deaths in patch i from this compartment

(10) New infections destined to be treated having arisen from susceptible populations in sub-patch 1 (local population) and sub-patch 2 (local population having returned from foreign travel) as these are infections due to local transmission.

(11) New infections destined to be treated having arisen from susceptible population with prior asymptomatic infection in sub-patches 1 and 2

(12) New clinical infections destined to remain untreated having arisen from susceptible populations in sub-patches 1 and 2

(13) New clinical infections destined to remain untreated having arisen from susceptible population with prior asymptomatic infection in sub-patches 1 and 2

(14) Development of clinical untreated infections to asymptomatic infections at a rate dependent on the duration of clinical untreated infections

(15) New asymptomatic infections destined to remain untreated having arisen from susceptible populations in sub-patches 1 and 2

(16) New asymptomatic infections destined to remain untreated having arisen from susceptible population with prior asymptomatic infection in sub-patches 1 and 2

(17) Development of asymptomatic to sub-patent infections at a rate dependent on the duration of asymptomatic infections

(18) Recovery of sub-patent infections at a rate dependent on the duration of sub-patent infections

Sub-patch 2 (Local population returning from foreign travel) : For each patch i moving to patch j $(i, j\ \epsilon\{1, 2, .., 6\}, j \neq i\ )$

$$\begin{aligned}
\frac{dS_{i,2}}{dt} &= -\underbrace{\lambda_i[t-\sigma]seas_i[t]S_{i,2}}_{(19)} + \frac{1}{r+\tau}I_{i,2} + \frac{1}{\rho}P_{i,2} - \underbrace{\frac{1}{\alpha}S_{i,2}}_{(20)} + \underbrace{\sum_j \frac{1}{\kappa_{i,j}}(S_{j,2}-S_{i,2})}_{(21)} \\
&\quad + \underbrace{\sum_j \frac{1}{\varpi_{i,j}}S_{j,3}}_{(22)} + \underbrace{\frac{1}{\zeta_{i,6}}S_{6,3}}_{(23)} - \mu S_{i,2} \\
\frac{dI_{i,2}}{dt} &= -\frac{1}{r+\tau}I_{i,2} - \frac{1}{\alpha}I_{i,2} + \sum_j \frac{1}{\kappa_{i,j}}(I_{j,2}-I_{i,2}) + \sum_j \frac{1}{\varpi_{i,j}}I_{j,3} + \frac{1}{\zeta_{i,6}}I_{j,3} - \mu I_{i,2} \\
\frac{dC_{i,2}}{dt} &= -\frac{1}{i_1}C_{i,2} - \frac{1}{\alpha}C_{i,2} + \sum_j \frac{1}{\kappa_{i,j}}(C_{j,2}-C_{i,2}) + \sum_j \frac{1}{\varpi_{i,j}}C_{j,3} + \frac{1}{\zeta_{i,6}}C_{j,3} - \mu C_{i,2} \\
\frac{dA_{i,2}}{dt} &= \frac{1}{i_1}I_{i,2} - \frac{1}{i_2}A_{i,2} - \frac{1}{\alpha}A_{i,2} + \sum_j \frac{1}{\kappa_{i,j}}(A_{j,2}-A_{i,2}) + \sum_j \frac{1}{\varpi_{i,j}}A_{j,3} + \frac{1}{\zeta_{i,6}}A_{j,3} - \mu A_{i,2} \\
\frac{dM_{i,2}}{dt} &= \frac{1}{i_2}A_{i,2} - \frac{1}{i_3}M_{i,2} - \frac{1}{\alpha}M_{i,2} + \sum_j \frac{1}{\kappa_{i,j}}(M_{j,2}-M_{i,2}) + \sum_j \frac{1}{\varpi_{i,j}}M_{j,3} + \frac{1}{\zeta_{i,6}}M_{j,3} - \mu M_{i,2} \\
\frac{dP_{i,2}}{dt} &= \frac{1}{i_3}M_{i,2} - \underbrace{\lambda_i[t-\sigma]seas_i[t](P_{i,2}+P_{i,2})}_{(24)} - \frac{1}{\rho}P_{i,2} - \frac{1}{\alpha}P_{i,2} + \sum_j \frac{1}{\kappa_{i,j}}(P_{j,2}-P_{i,2}) + \sum_j \frac{1}{\varpi_{i,j}}P_{j,3} \\
&\quad + \frac{1}{\zeta_{i,6}}P_{j,3} - \mu P_{i,2}
\end{aligned}$$

(19) New infections arising from sub-patch 2 due to local transmission and not infections contracted while travelling

(20) Assimilation of population in sub-patch 2 (locals having returned from foreign travel) back into sub-patch 1 from whence they originated.

(21) Movement between local patches (1-5) out of and into the compartment

(22) Movement of patch 6 population from foreign patch j, sub-patch 3 back into patch i but in sub-patch 2, when i=6 and j = 1-5; =0 for all other values of i as this rate is particular to movement of Maputo population (patch 6)

(23) Movement of patch i population from foreign patch 6 sub-patch 3 back into patch i, sub-patch 2, when i=1-5; =0 for j=6 as this rate is particular to movement of the Mpumalanga population to and from Maputo (patches 1-5)

(24) New infections arising from the susceptible population with a prior asymptomatic infection in sub-patch 2

Sub-patch 3 (Foreign population) : For each patch i moving to patch j $(i, j \,\epsilon\{1, 2, .., 6\}, j \neq i\ )$

$$\frac{dS_{i,3}}{dt} = -\underbrace{\lambda_i[t-\sigma]seas_i[t]S_{i,3}}_{(25)} + \frac{1}{r+\tau}I_{i,3} + \frac{1}{\rho}P_{i,3} + \underbrace{\sum_j \frac{1}{\kappa_{i,j}}(S_{j,3} - S_{i,3})}_{(26)} + \underbrace{\frac{1}{\varpi_{i,j}}(S_{j,1} - S_{i,3})}_{(27)}$$
$$+ \underbrace{\sum_j \frac{1}{\zeta_{i,j}}(S_{j,1} - S_{i,3})}_{(28)} - \mu S_{i,3}$$

$$\frac{dI_{i,3}}{dt} = \underbrace{pf_{yr}\lambda_i[t-\sigma]seas_i[t]S_{i,3}}_{(29)} + \underbrace{pf_{yr}\lambda_i[t-\sigma]seas_i[t]P_{i,3}}_{(30)} - \frac{1}{r+\tau}I_{i,3} + \sum_j \frac{1}{\kappa_{i,j}}(I_{j,3} - I_{i,3}) +$$
$$\frac{1}{\varpi_{i,j}}(I_{j,1} - I_{i,3}) + \sum_j \frac{1}{\zeta_{i,j}}(I_{j,1} - I_{i,3}) - \mu I_{i,3}$$

$$\frac{dC_{i,3}}{dt} = \underbrace{pc_1(1-pf_{yr})\lambda_i[t-\sigma]seas_i[t]S_{i,3}}_{(31)} + \underbrace{pc_2(1-pf_{yr})\lambda_i[t-\sigma]seas_i[t]P_{i,3}}_{(32)} - \frac{1}{i_1}C_{i,3}$$
$$+ \sum_j \frac{1}{\kappa_{i,j}}(C_{j,3} - C_{i,3}) + \frac{1}{\varpi_{i,j}}(C_{j,1} - C_{i,3}) + \sum_j \frac{1}{\zeta_{i,j}}(C_{j,1} - C_{i,3}) - \mu C_{i,3}$$

$$\frac{dA_{i,3}}{dt} = \underbrace{(1-pc_1)(1-pf_{yr})\lambda_i[t-\sigma]seas_i[t]S_{i,3}}_{(33)} + \underbrace{(1-pc_2)(1-pf_{yr})\lambda_i[t-\sigma]seas_i[t]P_{i,3}}_{(34)} + \frac{1}{i_1}C_{i,3} - \frac{1}{i_2}A_{i,3}$$
$$+ \sum_j \frac{1}{\kappa_{i,j}}(A_{j,3} - A_{i,3}) + \frac{1}{\varpi_{i,j}}(A_{j,1} - A_{i,3}) + \sum_j \frac{1}{\zeta_{i,j}}(A_{j,1} - A_{i,3}) - \mu A_{i,3}$$

$$\frac{dM_{i,3}}{dt} = \frac{1}{i_2}A_{i,3} - \frac{1}{i_3}M_{i,3} + \sum_j \frac{1}{\kappa_{i,j}}(M_{j,3} - M_{i,3}) + \frac{1}{\varpi_{i,j}}(M_{j,1} - M_{i,3}) + \sum_j \frac{1}{\zeta_{i,j}}(M_{j,1} - M_{i,3}) - \mu M_{i,3}$$

$$\frac{dP_{i,3}}{dt} = \frac{1}{i_3}M_{i,3} - \underbrace{\lambda_i[t-\sigma]seas_i[t]P_{i,3}}_{(35)} - \frac{1}{\rho}P_{i,3} + \sum_j \frac{1}{\kappa_{i,j}}(P_{j,3} - P_{i,3}) + \frac{1}{\varpi_{i,j}}(P_{j,1} - P_{i,3})$$
$$+ \sum_j \frac{1}{\zeta_{i,j}}(P_{j,1} - P_{i,3}) - \mu P_{i,3}$$

(25) New infections arising from sub-patch 3 due to local transmission and not infections contracted from patch of origin

(26) Movement between local patches (1-5) out of and into the compartment

(27) Movement of patch 6 population from patch 6, sub-patch 1 into patch i, sub-patch 3, when i=1-5 and j = 6 and movement from patch i, sub-patch 3 back into to patch 6, sub-patch 2. This rate =0 for all other values of j as it is particular to movement of Maputo population (patch 6)

(28) Movement of patch j population from patch j, sub-patch 1, into patch 6 sub-patch 3 when i=6 and j=1-5 and movement from patch 6, sub-patch 3 back into patch j, sub-patch 2 ; This rate=0 for j=6 as it is particular to movement of the Mpumalanga population (patches 1-5) to and from Maputo

(29) New infections destined to be treated having arisen from susceptible population in sub-patch 3

(30) New infections destined to be treated having arisen from susceptible population with prior asymptomatic infection in sub-patches 3

(31) New clinical infections destined to remain untreated having arisen from susceptible population in sub-patch 3

(32) New clinical infections destined to remain untreated having arisen from susceptible population with prior asymptomatic infection in sub-patch 3

(33) New asymptomatic infections destined to remain untreated having arisen from susceptible population in sub-patch 3

(34) New asymptomatic infections destined to remain untreated having arisen from susceptible population with prior asymptomatic infection in sub-patch 3

(35) New infections arising from the susceptible population with a prior asymptomatic infection in sub-patch 3

Table 1: Compartment Descriptions

Compartment	Description
$S_{i,k}$	Susceptible Population in patch i and sub-patch k
$I_{i,k}$	Infectious Treated Population in patch i and sub-patch k
$C_{i,k}$	Symptomatic Infectious Untreated Population in patch i and sub-patch k
$A_{i,k}$	Asymptomatic Infectious Untreated Population in patch i and sub-patch k
$M_{i,k}$	Infectious Population with Untreated Sub-patent Infectious in patch i and sub-patch k
$P_{i,k}$	Susceptible Population with prior asymptomatic infection in patch i and sub-patch k

Table 2: Values, descriptions and sources of the parameters driving the base metapopulation model of transmission. ($i = \{TC; MB; UJ; NK; BB; MP\}$)

Parameter	Description	Value	Source
N	Population size for the six patches	2.5×10^6	[2,3]
μ	Mortality/birth Rate	$\frac{105}{10000}$	[4]
σ	Period between liver stage and onset of gametocytemia	2 weeks	[5–8]
r	Artemether Lumefantrine elimination half-life	6 days	[9]
τ	Time to seek treatment	1/2 week	Expert opinion
ptf	Probability of treatment failure	0.01	[10]
p	Proportion of local infected population receiving treatment	0.95	[11,12]
pf_{yr}	Proportion of foreign infected population that receive treatment in a local patch	$pf_1 = 0.5851$ $(0.5850, 0.5853)$ (pre April 2005) $pf_2 = 0.7000$ $(0.6998, 0.7010)$ (post April 2005)	Estimated from model fitting process
i_1	Duration of clinical infection before becoming asymptomatic	0.7 weeks	[13]

i_2	Duration of asymptomatic infection before becoming sub-patent	5.5 weeks	[13,14]
i_3	Duration of sub-patent infection	24 weeks	[13]
ρ	Duration of clinical immunity	5 years	[15]
pc_1	Probability of clinical infection from naive individuals	0. 9997 (0.9756, 0.9999)	[7,16]
pc_2	Probability of clinical infection from partially immune individuals	0.883 (0.877, 0.888)	Estimated from data
$seas_i$	Seasonal forcing function for foreign sourced cases	Derived from data	[17]
β_i	Annual number of mosquito bites per person x proportion of bites testing positive for sporozoites for patch i	$\beta_{TC} = 4.488\ (4.178, 4.798)$<br>$\beta_{MB} = 6.034\ (5.967, 6.101)$<br>$\beta_{UJ} = 0.655\ (0.589, 0.723)$<br>$\beta_{NK} = 1.546\ (1.521, 1.571)$<br>$\beta_{BB} = 4.436\ (4.264, 4.609)$<br>$\beta_{MP} = 99.065\ (98.920, 99.210)$	Estimated from model fitting process
$\lambda_i(t)$	Force of infection	see Additional file 1	
$\frac{1}{\alpha}$	Rate of assimilation of population in sub-patch 2 (locals having returned from foreign travel) back into sub-patch 1 from whence they originated	1.5 weeks^{-1}	Expert opinion
$\frac{1}{k}$	Rate of movement between 5 Mpumalanga municipalities	1/ 201.436 (1/204.833, 1/198.040) weeks^{-1}	Estimated from model fitting process
$\frac{1}{v_{yr}}$	Maputo residents: Rate of movement between Maputo and 5 Mpumalanga municipalities	$\frac{1}{v_1} = 1/7616.743$ weeks^{-1} $(1/7663.186, 1/7570.299)$ (pre April 2005)<br>$\frac{1}{v_2} = 1/3227.213$ weeks^{-1} $(1/3187.684, 1/3266.742)$ (post April 2005)	Estimated from model fitting process
$\frac{1}{\varpi_{i,j}}$	Maputo residents: Rate of movement between Maputo and 5 Mpumalanga municipalities based on $\frac{1}{v_{yr}}$ and distance between patches	see Additional file 1	
$\frac{1}{z}$	Mpumalanga residents: Rate of movement between 5 Mpumalanga municipalities and Maputo	$\frac{1}{z} = 1/359.462$ weeks^{-1} (1/361.057, 1/357.866)	Estimated from model fitting process

$\frac{1}{\zeta_{i,j}}$	Mpumalanga residents: Rate of movement between 5 Mpumalanga municipalities and Maputo based on $\frac{1}{z}$ and distance between patches	see Additional file 1	
$fwgt$	Foreign movement weight intensity	10.615 (10.512, 10.719)	Estimated from model fitting process
$lwgt$	Local movement weight intensity	1.419 (1.343, 1.495)	Estimated from model fitting process
vef	Effectiveness of vector control	0.9785 (0.9783, 0.9787)	Estimated from model fitting process
$vc_i[t]$	Vector Control Coverage in patch i × efficiency	Derived from data	

Hybrid metapopulation DE-IBM model

The metapopulation DE model and the IBM model are linked such that the IBM model is nested in the DE model. At each time step, the DE model generates flows of a population that leave one compartment and enter another compartment (in the various sub-patches and patches). The IBM model takes the flow value at each time step once it has been negated from a compartment, discretises it into individuals in a population, executes the IBM algorithm, re-groups the individuals back into a population flow, and adds it into its destination compartment.

The metapopulation DE transmission model predicts (among other things) the number of local and foreign people entering the five Mpumalanga patches from the Maputo patch. These flows of the proportion of the population leaving compartments in the Maputo patch are interrupted before they enter the Mpumalanga patches. The proportion of the population is multiplied by the population size and discretised into individual people. These individual people are then subject to the individually-based FSAT model described earlier. The FSAT IBM algorithm is applied. Once all individuals have passed through the algorithm, they are grouped once again into populations per patch, sub-patch and compartment and the flow into Mpumalanga is completed and the transmission model continues processing as normal. For example, if the flow between Maputo and Nkomazi patch is 0.002 from the Susceptible compartment and 0.01 from the Infected compartment, then in a population of 1000 people, this translates to 2 susceptible individuals and 10 infected individuals passing through the border. Applying the FSAT IBM algorithm to these individuals may result in only 1 susceptible and 8 infected individuals agreeing to be screened. The

detectable threshold may be such that of the 8 infected individuals, only 6 test positive for malaria. Thus 6 receive treatment but perhaps only 4 adhere to the regimen and of the two that do not adhere to treatment, one fails treatment. In the absence of FSAT, two susceptible individuals and ten infected individuals would have been added to population of Nkomazi. The result of the FSAT campaign is that the 2 susceptible individuals remain malaria free, 5 of the 12 individuals are cured of their malaria and will be added to the susceptible population in Nkomazi. The 2 infected individuals who did not participate in the FSAT campaign and the one who failed treatment will be added to the infected population in Nkomazi. The 2 infected individuals who were screened but did not test positive will be added to the sub-patent infected population in Nkomazi. Thus the IBM model is completely nested in the metapopulation DE model.

Data Fitting Method

The model is fitted to weekly treated case data from 2002 to 2008, and then validated with data from 2009 to 2012. The model is run from 1990 to reach a steady state before being fitted to data from 2002. IRS coverage and drug treatment are included in the model for the data fitting. The number of treated cases in each sub-patch k are fitted to the data using the maximum likelihood approach assuming an underlying Poisson distribution with canonical parameter λ as the average number of treated cases per week. The metapopulation non-linear differential equation model is expressed in terms of average rates of movement between compartments, thus λ is a function of the parameters to be estimated (listed in Table 1)

The Poisson probability of observing x counts when the average number of counts per week is λ given by

$$P(x|\lambda) = \frac{\lambda^x \exp^{-\lambda}}{x!}.$$

As the model is being fitted to time series data with N time bins, λ, the expected number of counts per bin is a function of time. Assuming the independence of data from different time bins, the likelihood reduces to

$$L(\lambda_i|x_i) = \prod_{i=1}^{N} \frac{\lambda_i^{x_i} \exp^{-\lambda_i}}{x_i!}$$

and the log likelihood becomes

$$ln(L(\lambda_i|x_i)) = \sum_{i=1}^{N} x_i ln(\lambda_i) - \lambda_i - ln(x_i!).$$

The model is fitted to 16 sets of data for each weekly time bin: treated cases for three sub-patches in five Mpumalanga municipalities and treated cases for Maputo. Under the assumption of independence, the log likelihood to be maximised is

$$ln(L(\lambda_{s,i}|x_{s,i})) \propto \sum_{i=1}^{N}\sum_{s=1}^{16} x_{s,i} ln(\lambda_{s,i}) - \lambda_{s,i}$$

The log-likelihood is negated and minimised using the hydroPSO function implementing a version of the Particle Swarm Optimisation algorithm in the R package hydroPSO v0.3-3 [18, 19]. Particle Swarm Optimisation is a global stochastic optimisation technique initially inspired by social behaviour of birds and fish [20, 21]. It shares similarities with evolutionary optimisation techniques like Genetic Algorithms (GA) but explores the multi-dimensional solution space on the basis of individual and global best-known "particle positions" without evolution operators. Problems are optimised by moving particles (the population of candidate solutions) around the search-space based on the particles' position and velocity. Particle movements are a function of local best positions and other best particle positions in the search-space. Thus the particles "swarm" towards the best solutions in the search-space.

The parameters estimated through the model fitting process are presented in Table 1. The model with the estimated parameter values is run for a further 3 years to be further validated by comparison to data between 2009 and 2012. Figure 1 shows the data fitting and validation for all 16 sets of data. The model fits the data well with the exception of the Bushbuckridge patch pre 2006 in sub-patches 2 and 3, where the model overestimates the level of infections. This is to be expected as very little data was available on imported infections in this area at the time. Both the timing and level of malaria transmission in Maputo is captured by the model fitting process.

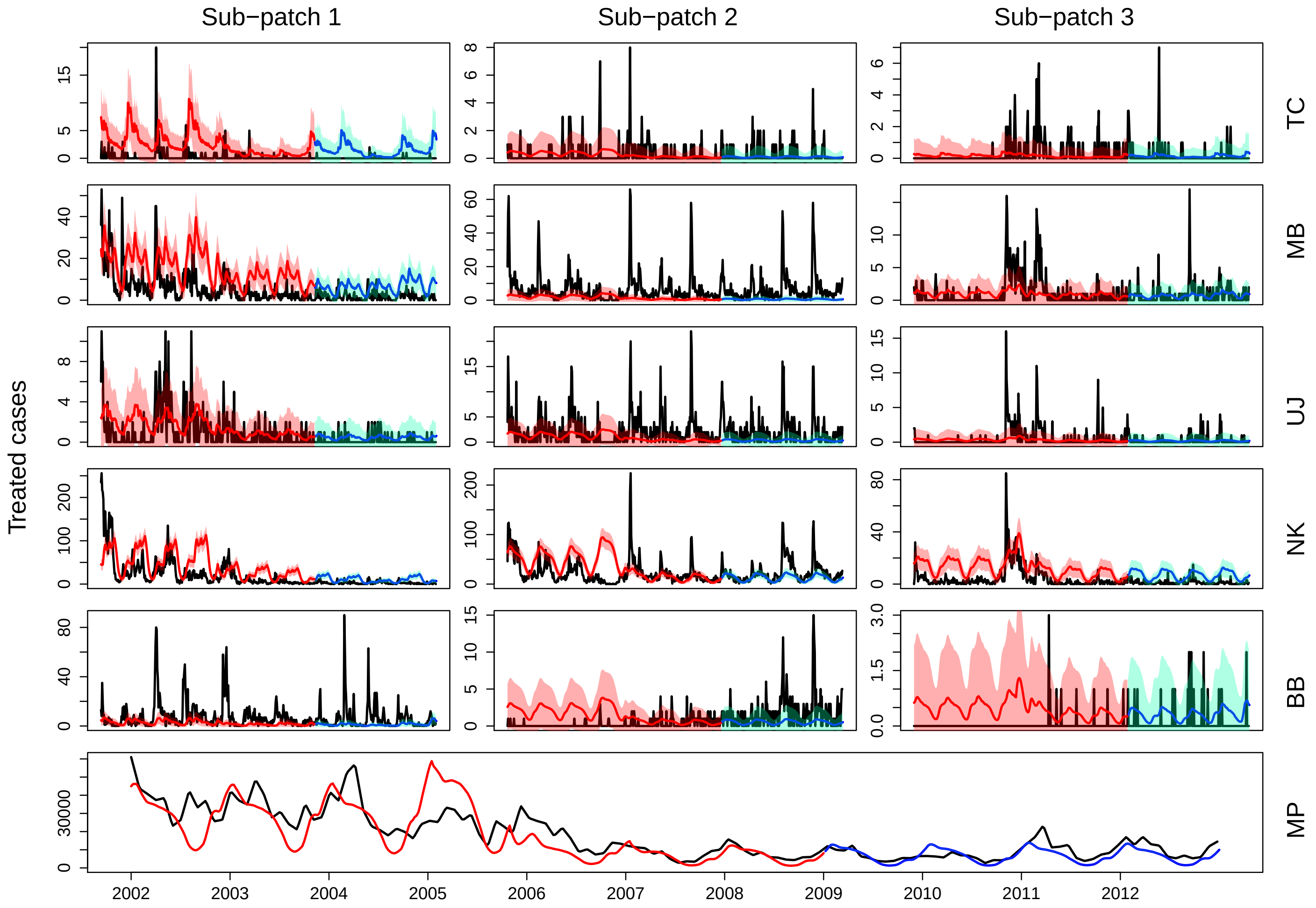

Figure 1: Predicted weekly treated cases (blue: 2002 - 2008; red: 2009-2012) fitted to and validated with data (black). The 95% uncertainty range for weekly case predictions is shown.

Test of Particle Swarm Optimisation routine

A synthetic test of the PSO routine is presented to assess if the method is capable of estimating the true model parameters and data given the high dimensionality of model. A set of parameters is prescribed for the model, and the model is run stochastically with this set of parameters to generate a dataset (Table 3). The optimisation routine is then performed on the model to assess if the search algorithm is able to locate the underlying parameter values. Figure 2 shows that the synthetic dataset closely resembles the deterministic model output generated with the true parameter values with a few noticeable differences: Malaria incidence in Nkomazi and Maputo are lower in the synthetic dataset, and rate of movement between residents of Maputo and Umjindi and Nkomazi are lower in the synthetic dataset. Figure 3 shows that the red line (model with estimated parameters from PSO routine) very closely resembles the synthetic dataset (black) and the estimated parameter values are in the region of the "true" parameter values (Table 3). As to be expected, the estimated β values for Nkomazi and Maputo are below the true values and the rate of movement from Maputo to the local municipalities is lower than the true value.

Table 3: Particle Swarm Optimisation Test Parameters

Parameter	Description	True	Estimated
β_i	Annual number of mosquito bites per person x proportion of bites testing positive for sporozoites for patch i	$\beta_{TC} = 4$<br>$\beta_{MB} = 6$<br>$\beta_{UJ} = 0.65$<br>$\beta_{NK} = 1.50$<br>$\beta_{BB} = 4$<br>$\beta_{MP} = 80$	$\beta_{TC} = 4.40$<br>$\beta_{MB} = 5.06$<br>$\beta_{UJ} = 0.68$<br>$\beta_{NK} = 1.19$<br>$\beta_{BB} = 3.08$<br>$\beta_{MP} = 65.53$
$\frac{1}{k}$	Rate of movement between 5 Mpumalanga municipalities	1/200	1/153.19
$\frac{1}{v_{yr}}$	Maputo residents: Rate of movement between Maputo and 5 Mpumalanga municipalities	$\frac{1}{v_1} = 1/7000$ (pre April 2005)<br>$\frac{1}{v_2} = 1/3000$ (post April 2005)	$\frac{1}{v_1} = 1/6953.23$ (pre April 2005)<br>$\frac{1}{v_2} = 1/3465.46$ (post April 2005)
$\frac{1}{z}$	Mpumalanga residents: Rate of movement between 5 Mpumalanga municipalities and Maputo	$\frac{1}{z} = 1/350$	$\frac{1}{z} = 1/300$
pc_2	Probability of clinical infection from partially immune individuals	0.80	0.793
pf	Proportion of foreign infected population that receive treatment in a local patch	0.60	0.55
$fwgt$	Foreign movement weight intensity	10	11.21
$lwgt$	Local movement weight intensity	1.5	1.25

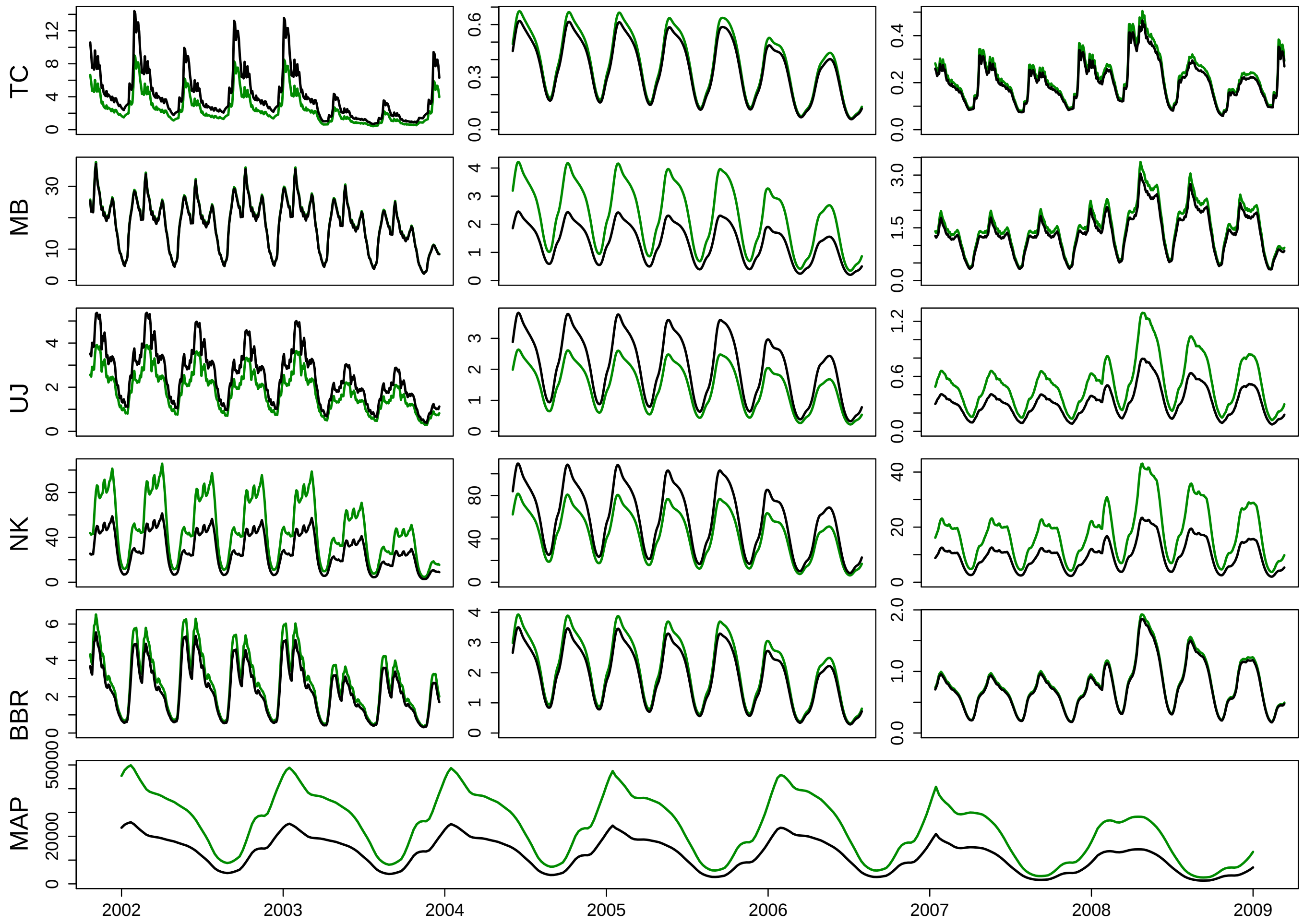

Figure 2: Predicted weekly treated cases for 2002-2008 for Model with true parameter values (green) and synthetic data generated from running this model stochastically (black)

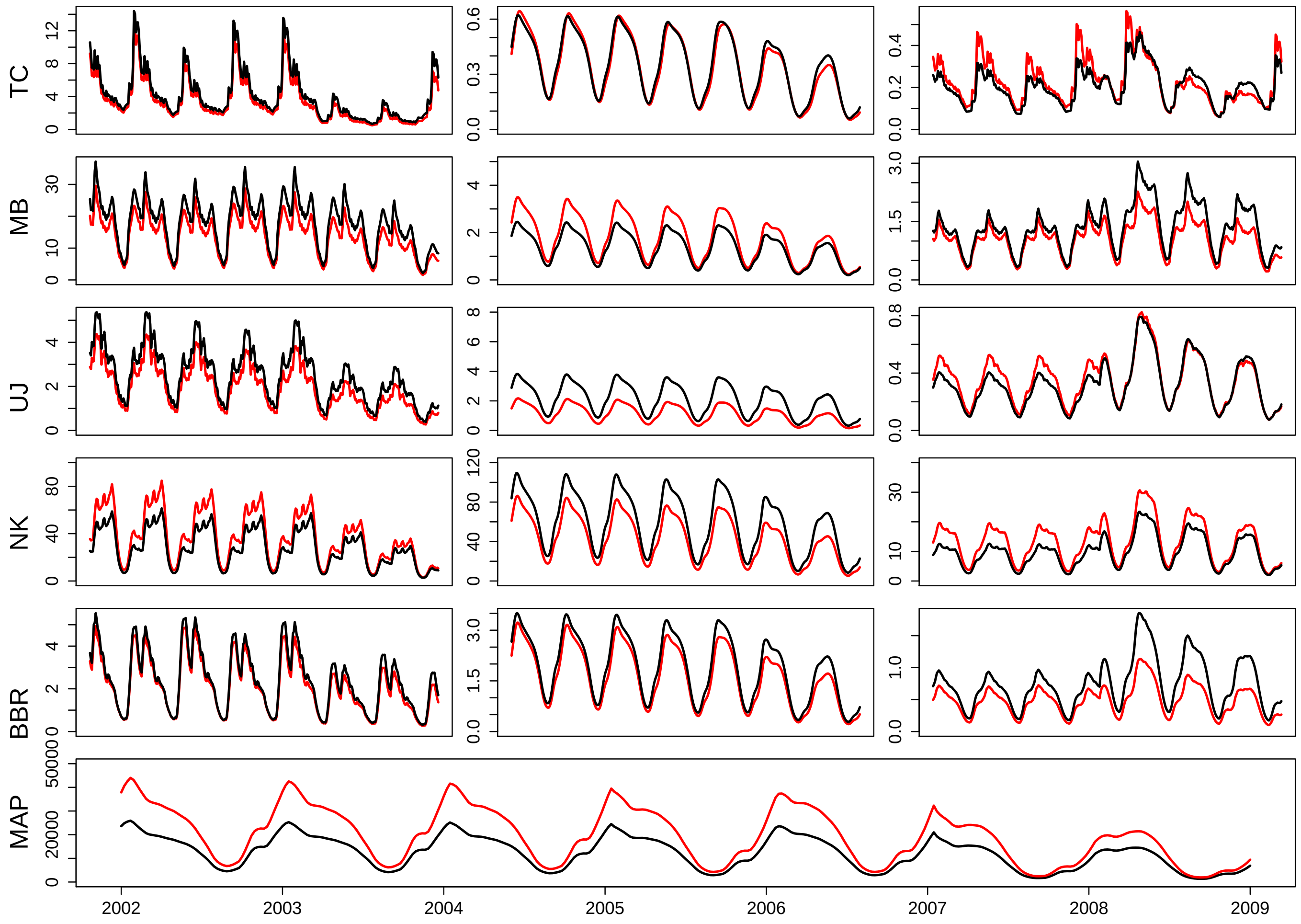

Figure 3: Predicted weekly treated cases for 2002-2008 for optimised model with estimated parameters (red) and synthetic data (black)

Migration rate sensitivity analysis

A test of the sensitivity of results to changes in the effect of varying coverage, detection thresholds, take-up proportions and adherence is presented for different levels of migration. As in the main paper, the decrease in local infections was measured for each combination of factors and a linear model regressing these four factors on the decrease in local infections was fitted to assess sensitivity. The absolute standardised regression coefficients in Figure 4 suggest that regardless of the level of migration and holding the other factors constant, detection threshold in an FSAT campaign has the largest absolute impact on decreasing local infections, followed by coverage achieved and take-up proportion.

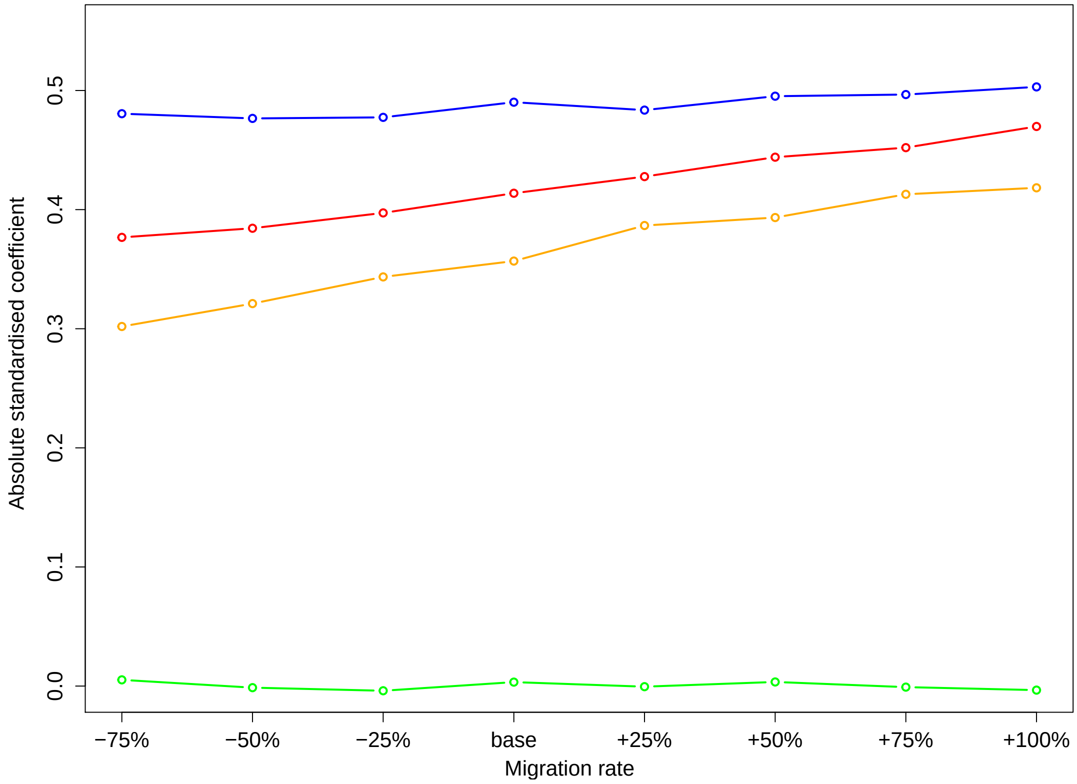

Figure 4: Sensitivity Analysis of factors assessed in FSAT model for different migration rates: detection threshold (blue), coverage (red), take-up proportion (orange) and adherence (green)

References

1. Silal SP, Little F, Barnes KI, White LJ: **Hitting a moving target: a model for malaria elimination in the presence of population movement**. *PLoS One* 2015, :*Under Review*.
2. **Census 2011 Municipal report Mpumalanga**. Tech. rep., Statistics South Africa 2012, [http://www.statssa.gov.za/Census2011/Products/MP_Municipal_Report.pdf].
3. Zacarias OP, Andersson M, et al.: **Mapping malaria incidence distribution that accounts for environmental factors in Maputo Province-Mozambique**. *Malar J* 2010, **9**:79.
4. **Mortality and causes of death in South Africa, 2010: Findings from death notification**. Tech. rep., Statistics South Africa, Pretoria 2013, [http://www.statssa.gov.za/publications/p03093/p030932010.pdf].
5. Thomson D: ***A Research Into the Production, Life and Death of Crescents in Malignant Tertian Malaria, in Treated and Untreated Cases, by an Enumerative Method; The Leucocytes in Malarial Fever: A Method of Diagnosing Malaria Long After it is Apparently Cured***. University Press 1911.
6. Eyles DE, Young MD: **The duration of untreated or inadequately treated *Plasmodium falciparum* infections in the human host.** *J Natl Malar Soc* 1951, **10**(4):327–36, [http://www.ncbi.nlm.nih.gov/pubmed/14908561].
7. Collins WE, Jeffery GM: **A retrospective examination of sporozoite- and trophozoite-induced infections with *Plasmodium falciparum*: development of parasitologic and clinical immunity during primary infection.** *Am J Trop Med Hyg* 1999, **61**(1 Suppl):4–19, [http://www.ncbi.nlm.nih.gov/pubmed/10432041].
8. Chitnis N, Hyman JM, Cushing JM: **Determining important parameters in the spread of malaria through the sensitivity analysis of a mathematical model.** *Bull Math Biol* 2008, **70**(5):1272–96, [http://www.ncbi.nlm.nih.gov/pubmed/18293044].
9. Makanga M, Krudsood S: **The clinical efficacy of artemether/lumefantrine (Coartem).** *Malar J* 2009, **8**(Suppl 1), [http://www.malariajournal.com/content/8/S1/S5].
10. Barnes KI, Durrheim DN, Little F, Jackson A, Mehta U, Allen E, Dlamini SS, Tsoka J, Bredenkamp B, Mthembu DJ, et al.: **Effect of artemether-lumefantrine policy and improved vector control on malaria burden in KwaZulu-Natal, South Africa**. *PloS Med* 2005, **2**(11):e330.
11. Castillo-Riquelme M, McIntyre D, Barnes K: **Household burden of malaria in South Africa and Mozambique: is there a catastrophic impact?** *Trop Med Int Health* 2008, **13**:108–22, [http://www.ncbi.nlm.nih.gov/pubmed/18291009].
12. Hlongwana KW, Zitha A, Mabuza AM, Maharaj R: **Knowledge and practices towards malaria amongst residents of Bushbuckridge, Mpumalanga, South Africa**. *Afr J Prim Health Care Fam Med* 2011, **3**, [http://www.phcfm.org/index.php/phcfm/article/view/257].
13. Griffin JT, Hollingsworth TD, Okell LC, Churcher TS, White M, Hinsley W, Bousema T, Drakeley CJ, Ferguson NM, Basáñez MG, Ghani AC: **Reducing *Plasmodium falciparum* Malaria Transmission in Africa: A Model-Based Evaluation of Intervention Strategies.** *PloS Med* 2010, **7**(8), [http://www.ncbi.nlm.nih.gov/pubmed/20711482].
14. Sama W, Owusu-Agyei S, Felger I, Dietz K, Smith T: **Age and seasonal variation in the transition rates and detectability of *Plasmodium falciparum* malaria.** *Parasitol* 2006, **132**(Pt 1):13–21, [http://www.ncbi.nlm.nih.gov/pubmed/16393349].
15. Filipe JAN, Riley EM, Drakeley CJ, Sutherland CJ, Ghani AC: **Determination of the processes driving the acquisition of immunity to malaria using a mathematical transmission model.** *PLoS Comput Biol* 2007, **3**(12):e255, [http://dx.plos.org/10.1371/journal.pcbi.0030255].
16. Griffin JT, Ferguson NM, Ghani AC: **Estimates of the changing age-burden of *Plasmodium falciparum* malaria disease in sub-Saharan Africa**. *Nat Commun* 2014, **5**.
17. Silal SP, Barnes KI, Kok G, Mabuza A, Little F: **Exploring the seasonality of reported treated malaria cases in Mpumalanga, South Africa.** *PLoS One* 2013, **8**(10):e76640, [http://www.plosone.org/article/info%3Adoi%2F10.1371%2Fjournal.pone.0076640].
18. Zambrano-Bigiarini, M, Rojas, R: **A model-independent Particle Swarm Optimisation software for model calibration**. *Environ Model Softw* 2013, **43**:5–25, [http://dx.doi.org/10.1016/j.envsoft.2013.01.004].

19. Zambrano-Bigiarini M, Rojas R: ***hydroPSO: Particle Swarm Optimisation, with focus on Environmental Models*** 2013, [http://www.rforge.net/hydroPSO,http://cran.r-project.org/web/packages/hydroPSO]. [R package version 0.3-3 — For new features, see the 'NEWS' file (on CRAN, rforge or the package source)].

20. Kennedy J, Eberhart R: **Particle swarm optimization**. In ***Proceedings of ICNN'95 - International Conference on Neural Networks***, *Volume 4*, IEEE:1942–1948, [http://ieeexplore.ieee.org/articleDetails.jsp?arnumber=488968].

21. Eberhart R, Kennedy J: **A new optimizer using particle swarm theory**. In ***MHS'95. Proceedings of the Sixth International Symposium on Micro Machine and Human Science***, IEEE 1995:39–43, [http://ieeexplore.ieee.org/articleDetails.jsp?arnumber=494215].
